# Host resistance to endotoxic shock requires the neuroendocrine regulation of group 1 innate lymphoid cells

**DOI:** 10.1084/jem.20171048

**Published:** 2017-12-04

**Authors:** Linda Quatrini, Elisabeth Wieduwild, Sophie Guia, Claire Bernat, Nicolas Glaichenhaus, Eric Vivier, Sophie Ugolini

**Affiliations:** 1 Aix-Marseille Université, CNRS, INSERM, CIML, Centre d'Immunologie de Marseille-Luminy, Marseille, France; 2 Université Côte d’Azur, CNRS, INSERM, Institut de Pharmacologie Moléculaire et Cellulaire, Valbonne, France; 3 Service d’Immunologie, Hôpital de la Timone, Assistance Publique-Hôpitaux de Marseille, Marseille, France

## Abstract

Upon infection, the immune system produces inflammatory mediators important for pathogen clearance. However, inflammation can also have deleterious effect on the host and is tightly regulated. Immune system–derived cytokines stimulate the hypothalamic–pituitary–adrenal (HPA) axis, triggering endogenous glucocorticoid production. Through interaction with ubiquitously expressed glucocorticoid receptors (GRs), this steroid hormone has pleiotropic effects on many cell types. Using a genetic mouse model in which the gene encoding the GR is selectively deleted in NKp46^+^ innate lymphoid cells (ILCs), we demonstrated a major role for the HPA pathway in host resistance to endotoxin-induced septic shock. GR expression in group 1 ILCs is required to limit their IFN-γ production, thereby allowing the development of IL-10–dependent tolerance to endotoxin. These findings suggest that neuroendocrine axes are crucial for tolerization of the innate immune system to microbial endotoxin exposure through direct corticosterone-mediated effects on NKp46-expressing innate cells, revealing a novel strategy of host protection from immunopathology.

## Abstract (precis)

Quatrini et al. demonstrate that neuroendocrine regulation of IFN-γ production by group 1 innate lymphoid cells (ILCs) is required to develop an IL-10–dependent resistance to endotoxin-induced septic shock, revealing a novel strategy of host protection from immunopathology.

## Introduction

Both the immune and nervous systems are involved in the maintenance of homeostasis and host integrity. Upon infection, a delicate balance is established between resistance mechanisms, which induce pathogen elimination, and tolerance mechanisms, which prevent excessive tissue damage (Medzhitov et al., 2012). It is becoming increasingly clear that neuroendocrine–immune interactions play an important role in these regulatory processes (Irwin and Cole, 2011), but the mechanisms involved are still not well understood.

IFN-γ is an important endogenous regulator of the immune response (Schroder et al., 2004). In bacterial infections, IFN-γ primes mononuclear phagocytes for phagocytosis and production of inflammatory cytokines promoting pathogen clearance (Schroder et al., 2004). These inflammatory processes are tightly regulated, and uncontrolled inflammation can induce clinical complications, such as septic shock. In particular, a process of tolerance to endotoxins has an important role for protecting the host against bacteria-induced shock (López-Collazo and del Fresno, 2013). This phenomenon is observed when exposure to low doses of endotoxins such as LPS, a major component of inflammation produced by gram-negative bacteria, reprograms the innate immune system, which becomes transiently more tolerant to subsequent high-dose endotoxin challenges. In experimental models of endotoxin tolerance, myeloid cells have been shown to switch to an antiinflammatory phenotype (Adib-Conquy et al., 2006; Foster et al., 2007; Porta et al., 2009; Yoza and McCall, 2011). This reprogramming of myeloid functions can be prevented by IFN-γ treatment both in vitro and in vivo (Mengozzi et al., 1991; Bundschuh et al., 1997; Chen and Ivashkiv, 2010). Interestingly, reduced IFN-γ production following the induction of endotoxin tolerance has been described in both humans and mice (Balkhy and Heinzel, 1999; Lauw et al., 2000; Varma et al., 2001). However, the mechanisms involved in the down-regulation of IFN-γ production and its consequences for host resistance to disease remain to be addressed.

The main cellular sources of IFN-γ immediately after pathogen invasion are group 1 innate lymphoid cells (ILCs; Chiche et al., 2011; Sonnenberg and Artis, 2015). These innate lymphocytes produce IFN-γ in response to various stimuli, including the inflammatory cytokines IL-12 and IL-18 released by myeloid cells (Varma et al., 2001). Group 1 ILCs comprise conventional natural killer (NK) cells, which are present in many organs, including the spleen, and circulate between the blood and tissues (Vivier et al., 2008). In addition, tissue-resident ILC1s that produce IFN-γ have been identified. In the liver, for instance, these cells share several markers with NK cells (such as NKp46 and NK1.1) and can be distinguished from conventional NK cells on the basis of the mutually exclusive expression of CD49a and CD49b (Peng et al., 2013; Yokoyama et al., 2013; Cortez and Colonna, 2016).

In addition to inducing an inflammatory response, LPS also indirectly activates the central nervous system. Indeed, LPS-induced IL-6, IL-1β, and TNF-α stimulate the hypothalamic–pituitary–adrenal (HPA) axis, which in turn induces the production of glucocorticoids (GCs; Rivier et al., 1989; Perlstein et al., 1993). In steady-state conditions, GCs (cortisol in humans and corticosterone in rodents) are released into the bloodstream by the adrenal gland according to a circadian rhythm regulated by the HPA axis. These steroid hormones optimize the synchronization of physiological and behavioral processes with the external environment (Dumbell et al., 2016; Oster et al., 2016). In conditions of inflammation, such as infection, that induce systemic cytokine production, GCs are among the principal effectors of the “stress response,” which results from an interaction between the neuroendocrine and immune systems responsible for maintaining physiological homeostasis (Cain and Cidlowski, 2017). GCs have long been recognized to have salient immunosuppressive and antiinflammatory functions and these properties have been widely exploited in clinical practice (Kadmiel and Cidlowski, 2013). However, understanding the role and mode of action of endogenously produced GCs is much more challenging (Cain and Cidlowski, 2017). Importantly, the disruption of the HPA axis through surgery (adrenalectomy) or pharmacological modulation (with GC receptor antagonists) renders mice more susceptible to septic shock because of the deleterious effects of hyperinflammation (Bertini et al., 1988; Edwards et al., 1991; Ramachandra et al., 1992). In addition, adrenalectomized mice cannot develop LPS tolerance, suggesting that endogenous GCs may regulate this process (Evans and Zuckerman, 1991). However, the cellular targets of GCs and the mechanisms involved remain elusive.

We used here a conditional knockout mouse model in which the gene encoding the glucocorticoid receptor is selectively deleted in NKp46^+^ innate lymphoid cells. This approach allowed us to investigate precisely how group 1 ILC functions are regulated by endogenous glucocorticoids and revealed the importance of this pathway for host resistance to endotoxin-induced systemic inflammatory disease.

## Results and discussion

### Tolerance to endotoxic shock is associated with high systemic GC levels

**Figure 1.**
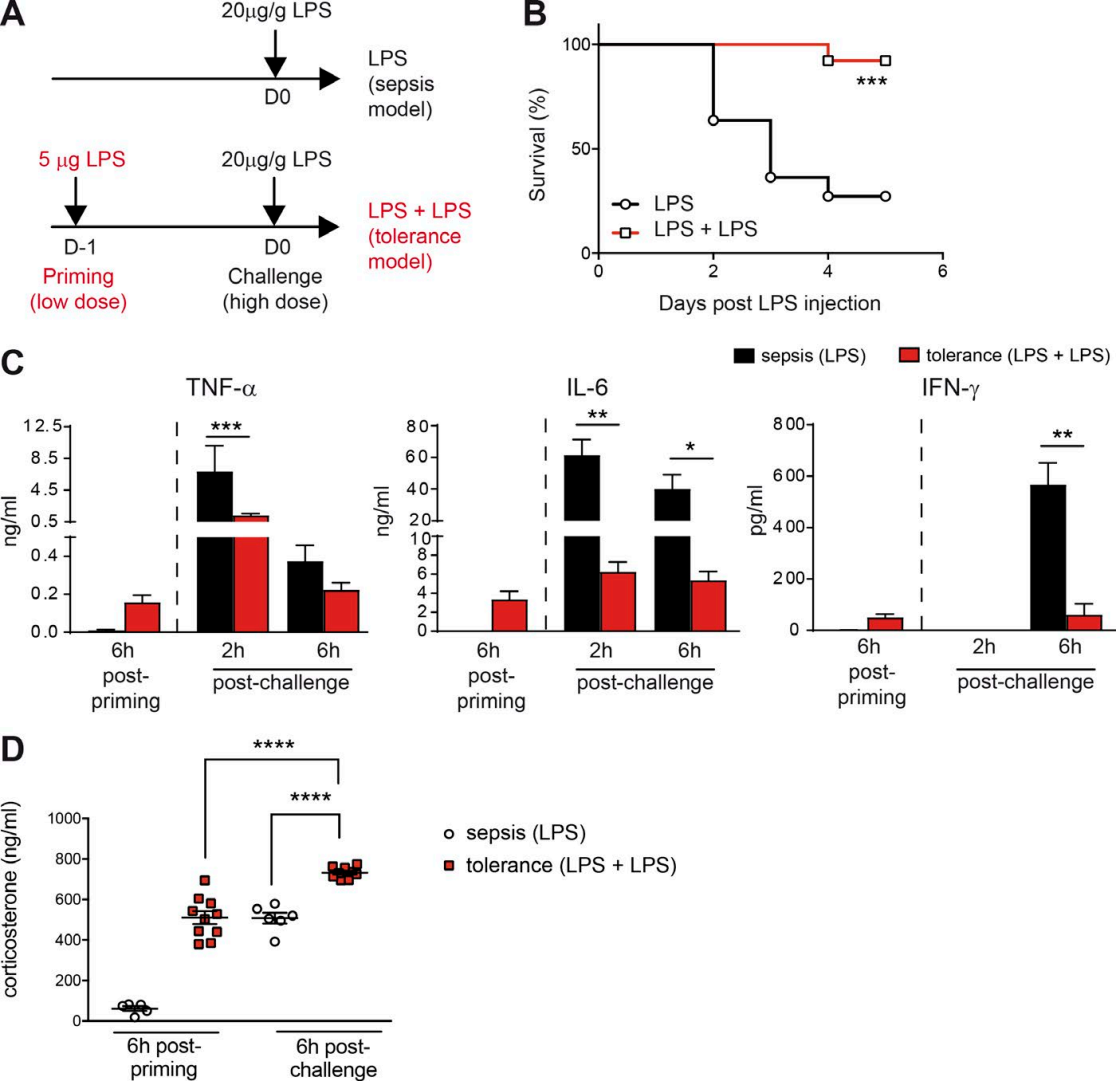
**Repeated LPS injections promote resistance to endotoxic shock and GC production.** (A) Protocol to induce endotoxin-induced sepsis or endotoxin tolerance. For the sepsis model, mice not injected or injected with PBS at day 1 (D-1) behave equally (not depicted). (B) Survival curve for mice receiving LPS injections according to the protocol in A. *n* = 11–12 mice pooled from two independent experiments; ***, P < 0.001 (Mantel-Cox test). (C and D) Cytokines and corticosterone concentrations in the serum. Data are presented as mean ± SEM. Each symbol in D represents a single mouse. *n* = 6–10 mice pooled from two independent experiments; *, P < 0.05; **, P < 0.01; ***, P < 0.001; ****, P < 0.0001 (one-way ANOVA).

Upon bacterial infections, the cytokines produced in the inflammatory cascade (including TNF-α, IL-1, IL-6, IL-12, and IFN-γ) are important for pathogen elimination, but they also have deleterious effects on the host. We studied the molecular and cellular mechanisms involved in the establishment of tolerance to endotoxin, by challenging WT C57BL/6 mice i.p. with LPS to mimic inflammation induced by a bacterial infection without the complexity associated with pathogen replication. In our model of endotoxin tolerance, the injection of a low dose of LPS into the mouse footpad to mimic peripheral infection is followed, 24 h later, by a systemic injection (i.p.) of a high dose of LPS (Fig. 1 A). We compared these primed mice with control mice receiving only a single high dose of LPS (model of septic shock). LPS priming significantly improved survival upon rechallenge (Fig. 1 B), and these tolerant mice had lower serum concentrations of the proinflammatory cytokines TNF-α, IL-6, and IFN-γ (Fig. 1 C), validating our in vivo model of endotoxin tolerance and consistent with previous studies.

We then investigated whether exposure to endotoxin differentially affected HPA axis activation by measuring GC production. The mechanisms underlying HPA axis activation in response to bacterial products have been described elsewhere (Rivier et al., 1989; Perlstein et al., 1993); IL-6, TNF-α, and IL-1 stimulate the hypothalamus to induce GC secretion (Dunn, 2000). A single injection of a low dose of LPS (used for priming) was sufficient to trigger the same level of corticosterone in the blood as a single injection of a high dose of LPS (challenge; Fig. 1 D). Thus, low doses of LPS used for priming in the tolerance model induced a systemic response stimulating the HPA axis comparably to a large dose of LPS used for the challenge in the sepsis model (Fig. 1 C). In addition, priming with a low dose of LPS significantly increased the amount of corticosterone produced after the subsequent injection of a high dose of LPS (Fig. 1 D). These data thus show that endotoxin tolerance is associated with high levels of GC production after both LPS priming and challenge, and they led us to dissect further the role of the HPA axis in the development of endotoxin tolerance.

### GR expression and function on NKp46^+^ ILCs

The GC receptor (GR; encoded by the nuclear receptor subfamily 3 group C member 1 gene *Nr3c1*) is an ubiquitously expressed transcription factor. It can regulate many different gene networks, depending on particular cellular and physiological contexts, making it difficult to precisely dissect the mechanisms involved in a given physiological process (Weikum et al., 2017). This complexity can be circumvented by addressing its role in specific cell types. In the LPS-induced septic shock model, previous studies have shown that GCs can inhibit the expression of inflammatory cytokines by specifically acting on their cellular sources; such mechanisms have been shown to operate for the production of IL-1β, TNF-α, and IL-6 by monocytes and macrophages (Bhattacharyya et al., 2007; Kleiman et al., 2012) and of IL-12 by dendritic cells (DCs; Li et al., 2015). Because IFN-γ is a central mediator of inflammation that is down-regulated in endotoxin tolerance models (Varma et al., 2001), we focused our study on group 1 ILCs, which are characterized by their capacity to produce IFN-γ. We investigated the regulatory mechanisms involved and the role of IFN-γ production in these inflammatory conditions by generating a conditional knockout mutant of the *Nr3c1* gene to eliminate the GR specifically in NKp46^+^ cells. We crossed *Ncr1^iCre^* mice (expressing an “improved” Cre [iCre] recombinase from the locus encoding NKp46; Narni-Mancinelli et al., 2011) with mice in which the third exon of the *Nr3c1* gene encoding GR was flanked by loxP recombination sites (*Nr3c1^LoxP/LoxP^*; Tronche et al., 1999) to generate *Ncr1^iCre/+^Nr3c1^LoxP/LoxP^* mice (referred to hereafter as GR*^Ncr1-iCre^* mice). In this study, *Ncr1^iCre/+^* littermates were used as controls (referred to hereafter as control mice).

**Figure 2.**
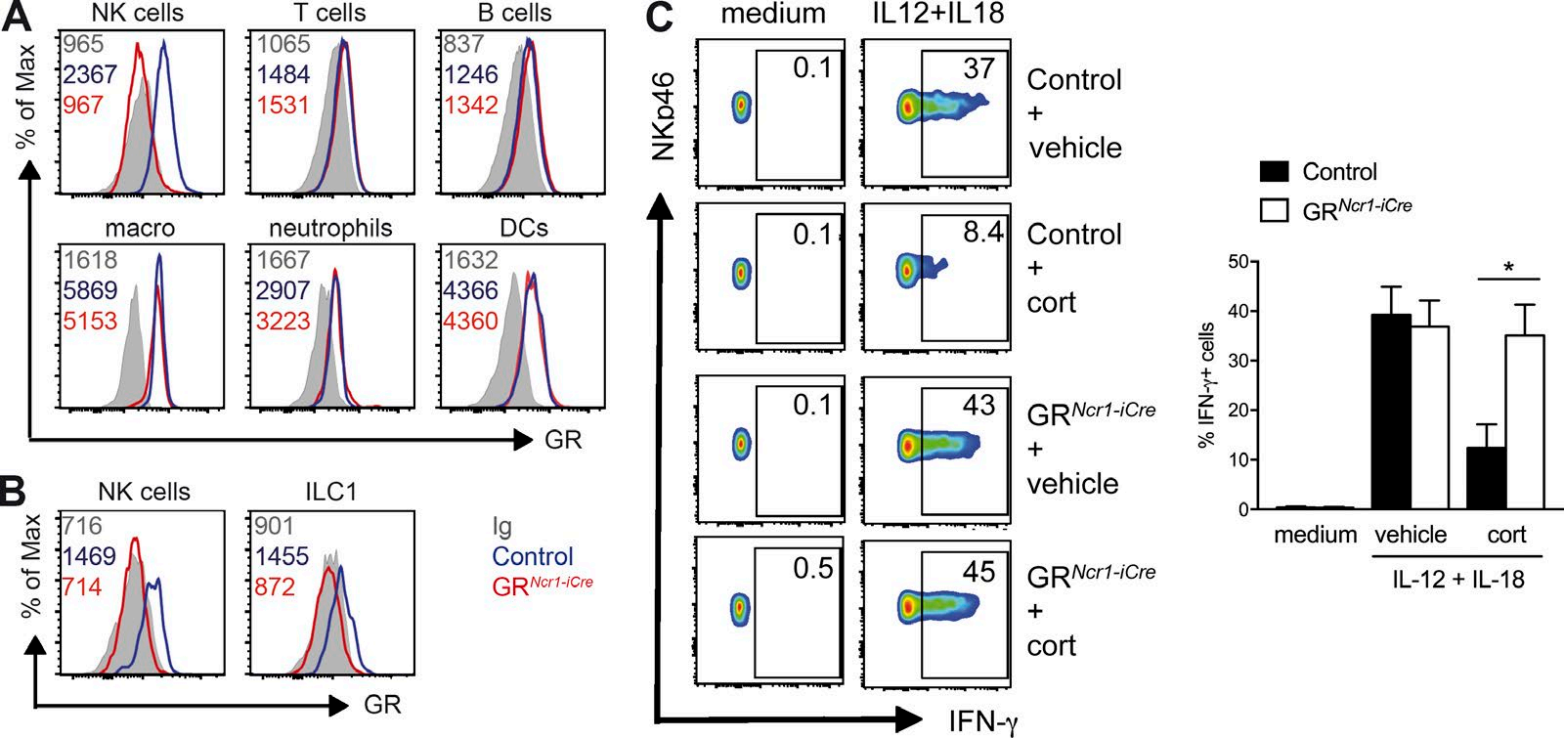
**Corticosterone inhibits IFN-γ production by NK cells through GR signaling.** (A and B) GR expression and isotype control (Ig) in NK cells (NKp46^+^NK1.1^+^CD3^−^CD19^−^), T cells (CD3^+^NK1.1^−^CD19^−^), B cells (CD19^+^CD3^−^), macrophages (CD11b^+^F4/80^+^), neutrophils (CD11b^+^Ly6G^+^), and DCs (CD11c^+^MHCII^+^) from the spleen (A) and NK cells (NKp46^+^NK1.1^+^CD3^−^CD19^−^DX5^+^CD49a^−^) and resident ILC1s (NKp46^+^NK1.1^+^CD3^−^CD19^−^DX5^−^CD49a^+^) in the liver (B). The numbers on the plots represent the mean fluorescence intensity. The data shown are representative of two independent experiments with five mice per group. (C) Splenocytes were left untreated (medium) or stimulated for 4 h with IL-12 and IL-18 in the presence of 500 nM corticosterone (cort) or vehicle alone. The percentage of IFN-γ^+^ NKp46^+^ cells is indicated for each FACS plot. Histograms represent these percentages as means + SEM (*n* = 7–8 mice pooled from three independent experiments; *, P < 0.05; Mann–Whitney *U* test).

We observed that splenic NKp46^+^ ILCs, most of which are conventional NK cells, expressed higher levels of GR than T and B lymphocytes (Fig. 2 A). In the liver, both NK cells and ILC1s also expressed GR (Fig. 2 B). An analysis of NKp46^+^ ILCs subsets in the small intestine showed that GR was expressed in NK cells, ILC1s, and the NKp46^+^ subset of ILC3s (not depicted). As expected, flow cytometry analysis revealed that the GR protein was selectively depleted in all NKp46^+^ ILCs subsets from the spleen (Fig. 2 A), liver (Fig. 2 B), and small intestine (not depicted) of GR*^Ncr1-iCre^* mice, whereas it was retained in other hematopoietic cells, including T cells, B cells, macrophages, neutrophils and DCs (Fig. 2 A).

We then analyzed the effect of the *Nr3c1* gene deletion in NKp46^+^ cells at steady-state. The numbers and percentages of all NKp46^+^ ILC subsets in the spleen, the liver and the small intestine of GR*^Ncr1-iCre^* mice were normal (Fig. S1 A and not depicted). In addition, the expression of the inhibitory and activating receptors Ly49G2, NKG2A, KLRG1, Ly49D, Ly49H and the maturation markers CD11b, CD27, CD43 revealed no change in NK cell maturation and phenotype (Fig. S1, B–D). These results demonstrate the lack of requirement of the GC–GR pathway for the development, maturation, and homeostasis of NK cells, ILC1s, and NKp46^+^ ILC3s at steady state, contrary to T cells, in which such signaling is required for thymocyte selection (Mittelstadt et al., 2012).

We then investigated the modulation of NK cell functions by GCs in an in vitro activation assay in the presence or absence of corticosterone. Corticosterone treatment inhibited IFN-γ production by spleen NK cells stimulated with IL-12 and IL-18 (Fig. 2 C). Importantly, this regulation was NK cell intrinsic, as it was not observed with splenocytes from GR*^Ncr1-iCre^* mice (Fig. 2 C).

These in vitro results highlighted the potential role of GR in NK cells in inflammatory conditions, prompting us to investigate whether the HPA axis, via the production of corticosterone, could modulate IFN-γ–producing ILC functions in vivo.

### The regulation of IFN-γ production associated with endotoxin tolerance requires GR expression in group 1 ILCs

**Figure 3.**
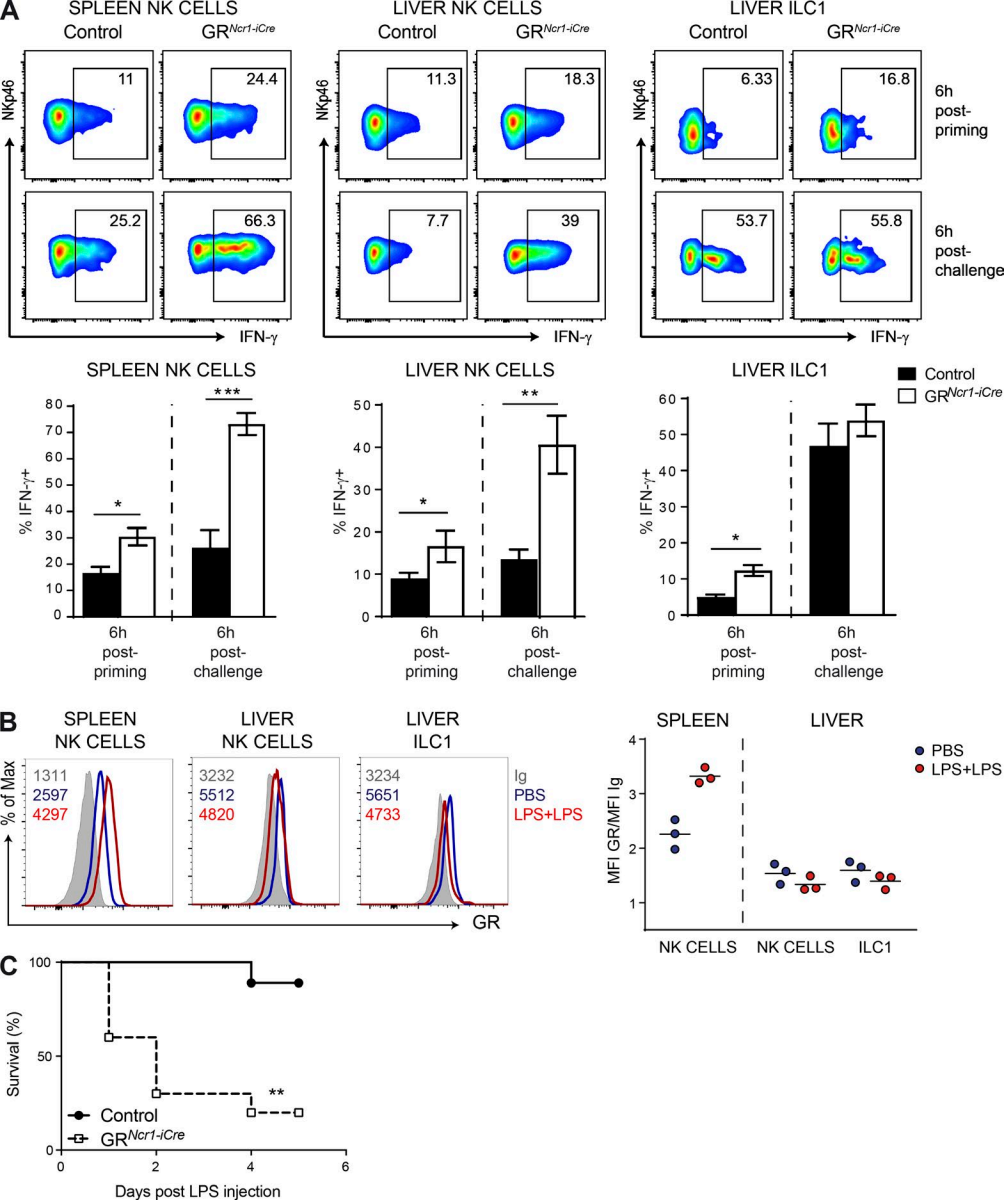
**Spleen and liver group 1 ILC responsiveness to GCs is required for the development of endotoxin tolerance.** (A) Percentage of IFN-γ^+^ NK cells (NKp46^+^NK1.1^+^CD3^−^CD19^−^DX5^+^CD49a^−^) and ILC1s (NKp46^+^NK1.1^+^CD3^−^CD19^−^DX5^−^CD49a^+^) in the spleen and liver of control and GR*^Ncr1-iCre^* mice, as determined 6 h after LPS priming and challenge. Data are presented as representative FACS plots and as mean ± SEM (*n* = 5–7 mice pooled from two independent experiments; *, P < 0.05; **, P < 0.01; ***, P < 0.001; Mann–Whitney *U* test). (B) GR expression and isotype control (Ig) measured 6 h after PBS injection or LPS challenge. FACS histograms and the mean value of the GR/Ig ratio from one representative experiment with three mice per group are shown. Each symbol represents a single mouse. (C) Survival curve for control and GR*^Ncr1-iCre^* mice (*n* = 7–11 mice pooled from two experiments; **, P < 0.01; Mantel–Cox test).

The mechanisms involved in the reduced IFN-γ production after the induction of endotoxin tolerance are poorly understood. We assessed the in vivo consequences of the modulation of NKp46^+^ ILC functions by endogenous GCs by challenging GR*^Ncr1-iCre^* mice and their control littermates using the endotoxin tolerance protocol. Six hours after LPS priming, we found that the frequency of splenic and liver NK cells and liver-resident ILC1s producing IFN-γ in GR*^Ncr1-iCre^* mice was much higher than that in control mice (Fig. 3 A). Moreover, 6 h after the secondary LPS injection (LPS challenge) the frequency of IFN-γ^+^ NK cells in both the spleen and liver of GR*^Ncr1-iCre^* mice was higher than that in the controls, whereas the production of IFN-γ by liver-resident ILC1s was unaffected at this time point (Fig. 3 A). Thus, during the priming phase, endogenous corticosterone inhibited IFN-γ production by NK cells and ILC1s. In contrast, upon secondary challenge, only spleen and liver NK cells had higher levels of IFN-γ production in the absence of GR signaling.

We then investigated the roles of the GR on group 1 ILC functions during the primary and secondary phases by determining whether GR expression was modulated during these inflammatory challenges. We found that multiple LPS challenge increased GR expression in spleen NK cells but had no effect or slightly decreased GR expression in liver NK cells and resident ILC1s (Fig. 3 B). The regulation of GR expression in NK cells in this model is, therefore, organ specific. Our results also suggest that the level of GR expression in ILC1s is not sufficient to account for the lack of regulation of IFN-γ production by the GC–GR pathway upon the secondary LPS challenge (although it may contribute to this effect), as liver NK cells, which have a similar phenotype, are sensitive to this regulation. This difference in ILC1 sensitivity to GR regulation between the two phases indicates that the mechanisms of control of IFN-γ production in these cells differs from that in NK cells in conditions of endotoxin tolerance.

As mentioned in the previous section, the NKp46^+^ innate cells targeted with the *Ncr1^iCre^* model include NK cells, ILC1s, and NKp46^+^ ILC3s (also called NCR^+^ ILC3s), which are undetectable in spleen and liver at steady state but present in the gut (Narni-Mancinelli et al., 2011). Upon LPS challenge, no IFN-γ production by small intestine RORγt^+^ ILC3s, NK1.1^+^RORγt^−^ Eomes^−^ ILC1s, and NK1.1^+^RORγt^−^ Eomes^+^ NK cells was detected ex vivo (not depicted), suggesting that these intestinal cell subsets do not play a major role in IFN-γ production in this model. As a control, we induced IFN-γ production in small-intestine NK cells and ILC1s (but not NCR^+^ ILC3s) upon in vitro restimulation with cytokines (IL-12 and IL-18), PMA, and ionomycin, showing that these cells can be responsive (Fig. S2 A). However, under these in vitro experimental conditions, the frequency of IFN-γ^+^ cells observed after the stimulation of cells from mice treated with PBS or LPS was similar, demonstrating that the responsiveness of these cells was not affected by the inflammatory or endogenous GC responses induced by the endotoxin (Fig. S2 A). We can thus conclude that NKp46^+^ cells in the small intestine are not a significant source of IFN-γ in our system.

We then checked whether parameters other than IFN-γ production were regulated by GR in group 1 ILCs by analyzing the TNF-α production of these cells, their proliferation, and their expression of other functional markers. The frequency of TNF-α-producing NK cells and ILC1s after LPS challenge was unchanged in the spleen and liver of GR*^Ncr1-iCre^* mice, as shown by comparison with control animals (Fig. S2 B). In addition, the absolute number of NK cells and ILC1s and their proliferative status, analyzed by evaluating the levels of the Ki67 marker, were similar in GR*^Ncr1-iCre^* and control mice (Fig. S2 C). Finally, levels of the activation markers CD69 and granzyme B were also similar (Fig. S2 D), demonstrating that, in the context of endotoxin tolerance, GR expression has no effect on these other parameters linked to cell activation and functional competence. Thus IFN-γ production is the only one of the “canonical” NK cell and ILC1 activation pathways analyzed to display selective sensitivity to GR regulation.

### NKp46^+^ cell responsiveness to GCs is required for the development of endotoxin tolerance

We evaluated the physiological relevance of the regulation of IFN-γ production in group 1 ILCs by the GR pathway by comparing the survival of GR*^Ncr1-iCre^* mice and their control littermates after LPS challenge. We found that priming with a low dose of LPS did not rescue survival in GR*^Ncr1-iCre^* mice, in marked contrast with control animals (Fig. 3 C), showing that the responsiveness of NKp46-expressing cells to corticosterone was required for the development of endotoxin tolerance.

In vivo studies in animal models (Delano et al., 2007) and ex vivo experiments on human peripheral blood mononuclear cells (Pena et al., 2011) have shown that multiple challenges with LPS induce myeloid cells to switch to an anti-inflammatory phenotype. Several tolerization mechanisms have been reported to operate at the signal transduction, transcriptional, and epigenetic levels (Mengozzi et al., 1991; Frankenberger et al., 1995; Foster et al., 2007). Interestingly, some of these mechanisms can be abolished by IFN-γ (Frankenberger and Ziegler-Heitbrock, 1997; Chen and Ivashkiv, 2010; Turrel-Davin et al., 2011). In particular, pretreatment with IFN-γ in vitro can prevent the tolerization of primary human monocytes and restore the TLR4-mediated induction of various proinflammatory cytokines, including IL-6 and TNF-α (Chen and Ivashkiv, 2010).

**Figure 4.**
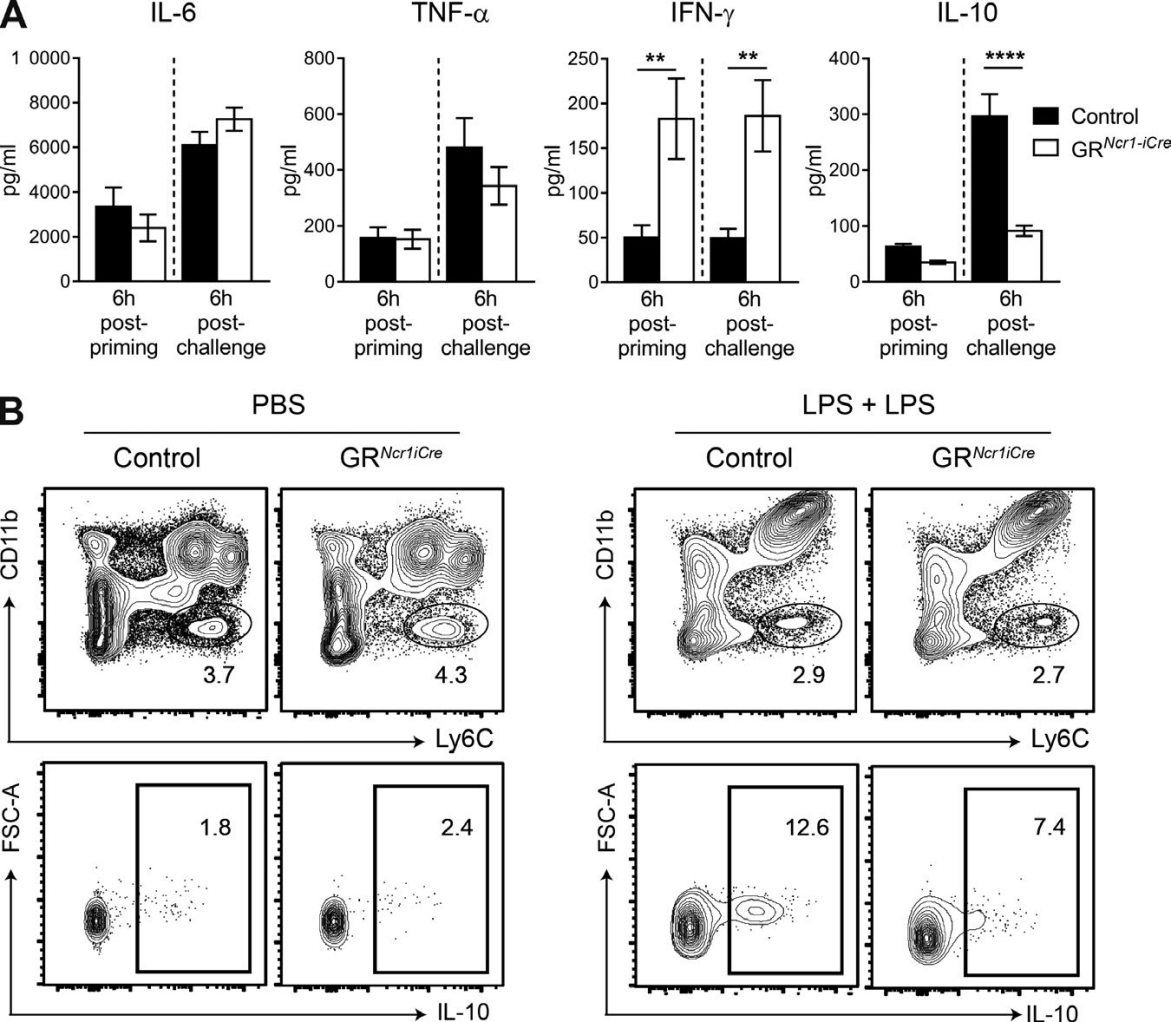
**Higher levels of IFN-γ in GR*^Ncr1-iCre^* mice are associated with lower systemic IL-10 concentrations.** (A) Cytokines in the serum, as determined 6 h after priming and after challenge with LPS. Data are presented as mean ± SEM (*n* = 14–16 mice pooled from three experiments; **, P < 0.01; ****, P < 0.0001; Student’s *t* test). (B) FACS plots showing IL-10 intracellular staining in Ly6C^+^CD11b^low^ cells 6 h after challenge with LPS, after gating out CD19^+^, CD3^+^, and CD11c^+^ cells. The data shown are representative of two independent experiments with seven mice per group.

We investigated the mechanisms by which this HPA-driven regulation of NKp46^+^ cells promotes endotoxin tolerance by determining the levels of inflammatory cytokines in the serum 6 h after the first (priming) and the second (challenge) LPS injections. In this in vivo model, we found that serum IL-6 and TNF-α concentrations were not significantly affected in GR*^Ncr1-iCre^* mice, despite the higher serum levels of IFN-γ in GR*^Ncr1-iCre^* mice both after priming and after rechallenge (Fig. 4 A). In contrast, IL-10 serum levels were similar after priming but significantly lower in these GR*^Ncr1-iCre^* animals after LPS rechallenge (Fig. 4 A). These results suggest that the down-regulation of IFN-γ production by corticosterone in group 1 ILCs has a major impact on systemic IFN-γ levels during both the priming and challenge phases. Moreover, a GR-dependent pathway in NKp46-expressing cells allows the development of an antiinflammatory status associated with the production of IL-10.

The reduction of systemic IL-10 levels in GR conditional KO mice was not caused by an intrinsic effect of GCs on IL-10 production by NKp46^+^ cells because these cells do not produce this cytokine (Fig. S3 A). Similarly, we did not detect IL-10 production in T cells, B cells, or DCs upon LPS challenge (not depicted). In contrast, we identified a population of Ly6C^high^ CD11b^low^ myeloid cells in the spleen as the main source of IL-10 in this model (Fig. 4 B). These data therefore suggest that microbial LPS exposure induces a direct corticosterone-mediated effect on NKp46-expressing group 1 ILCs, inhibiting their IFN−γ production. In addition, after a second encounter with endotoxin an indirect effect on the production of IL-10 by myeloid cells is observed.

### Corticosterone allows the development of IL-10–dependent endotoxin tolerance by preventing IFN-γ production by group 1 ILCs

**Figure 5.**
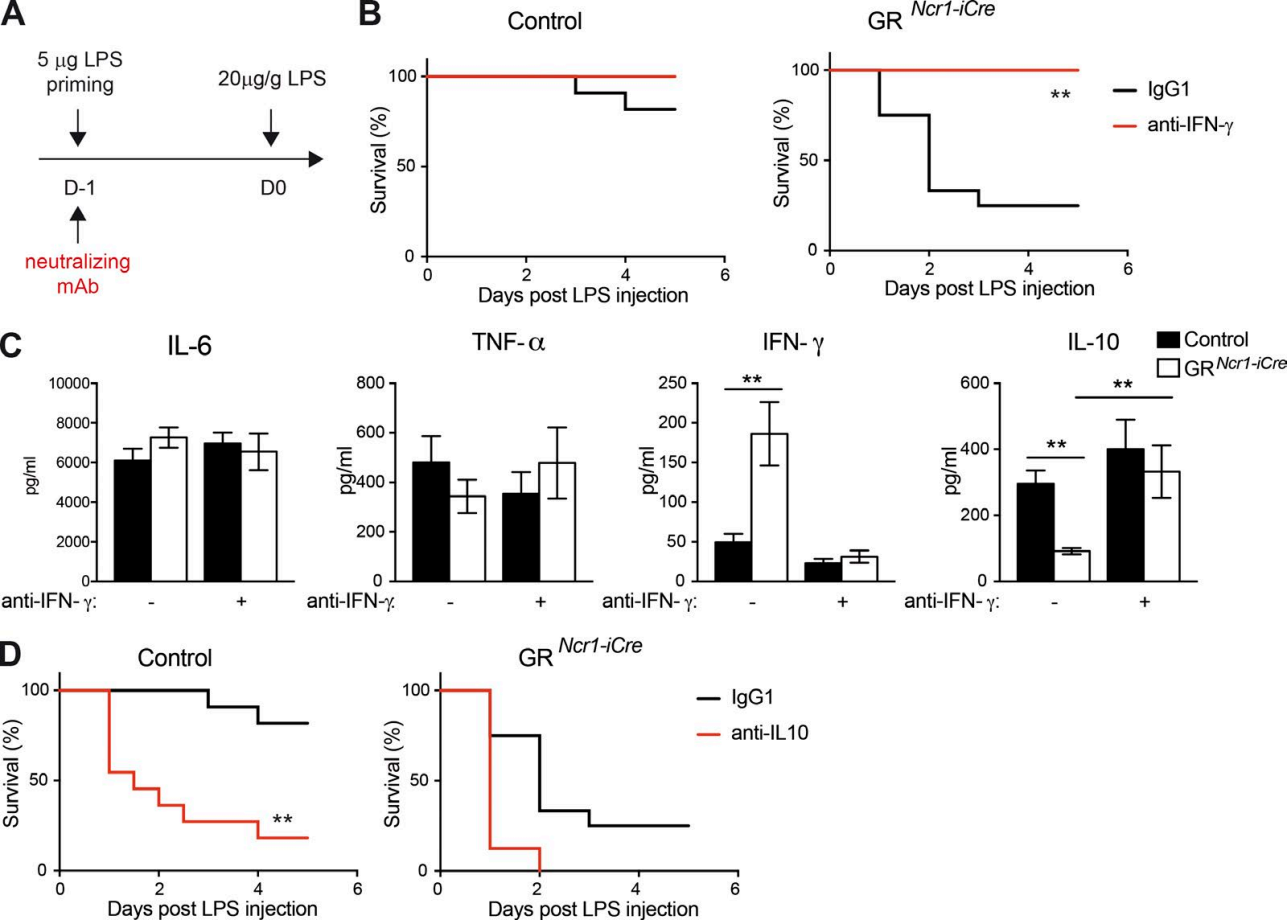
**IFN-γ neutralization restores tolerance to endotoxin and IL-10 production in GR*^Ncr1-iCre^* mice.** (A, B, and D) Mice received LPS injections and were treated with anti–IFN-γ (B) or anti–IL-10 (D) neutralizing antibodies or isotype control (IgG1) antibodies at the priming phase. (B) Survival curves for control and GR*^Ncr1-iCre^* mice treated with anti–IFN-γ neutralizing antibodies (*n* = 8–11 mice from two independent experiments, plotted in separated graphs; **, P < 0.01; Mantel–Cox test). (C) Cytokines in the serum as determined 6 h after challenge with LPS. Data are presented as mean ± SEM (*n* = 8–14 mice pooled from three independent experiments; **, P < 0.01; one-way ANOVA). (D) Survival curves for control and GR*^Ncr1-iCre^* mice treated with anti–IL-10 neutralizing antibodies (*n* = 8–11 mice from two independent experiments, plotted on separate graphs; **, P < 0.01; Mantel–Cox test).

The regulation by GCs of IFN-γ production by group 1 ILCs may therefore be crucial to maintain the correct balance between resistance to pathogens and resistance to inflammatory disease and immunopathology. We directly addressed the question of the role of the regulation of IFN-γ production by group 1 ILCs by the HPA axis in mouse resistance to disease, by challenging GR*^Ncr1-iCre^* and control animals with LPS in the presence or absence of monoclonal antibodies (mAbs) neutralizing IFN-γ during the priming phase (Fig. 5 A). In the presence of IFN-γ blockade, tolerance to endotoxin in GR*^Ncr1-iCre^* mice was rescued to levels similar to those observed in control mice (Fig. 5 B). Importantly, serum IL-10 concentration in GR*^Ncr1-iCre^* mice was also restored to control levels, confirming that the down-regulation of this cytokine was not intrinsically regulated in NKp46^+^ cells but extrinsically linked to the higher levels of IFN-γ (Fig. 5 C). In contrast, in accordance with the lack of change in TNF-α and IL-6 production levels in GR*^Ncr1-iCre^* mice upon endotoxin tolerance (Fig. 4 A), IFN-γ neutralization did not affect the production of these cytokines after LPS challenge (Fig. 5 C). Endogenous GCs produced after a first low-level microbial exposure thus regulate an IFN-γ–IL-10 axis, which is crucial for the establishment of endotoxin tolerance and host survival upon a secondary pathogen encounter. GR signaling in NKp46-expressing ILCs is essential for the regulation of this pathway. Interestingly, in the sepsis model (only one high dose of LPS injected), despite an increase in the systemic levels of IFN-γ in GR*^Ncr1-iCre^* mice, the amount of IL-10 was similar to that in the control (Fig. S3 B), suggesting that the reprograming of myeloid cell functions by IFN-γ is dependent on the inflammatory context.

Finally, the importance of the corticosterone regulation of group 1 ILC IFN-γ production and the consequent increase in systemic IL-10 levels was further demonstrated by IL-10 neutralization (Fig. S3 C), which abolished protection in control mice, reducing survival to a level similar to that in GR*^Ncr1-iCre^* mice (Fig. 5 D). The role of IL-10 in endotoxin tolerance has been a matter of debate. Some studies have reported a weak effect of this cytokine (Berg et al., 1995; Murphey and Sherwood, 2006), whereas others have reported a requirement for systemic IL-10 in endotoxin tolerance (Randow et al., 1995; Sfeir et al., 2001; Muehlstedt et al., 2002; Muenzer et al., 2010). It is possible that, in some specific experimental settings, the expression of other antiinflammatory factors (such as TGF-β) contributes to endotoxin tolerance. However, our data are consistent with a major role of IL-10 in this process, as we demonstrated that IL-10 neutralization completely abolished endotoxin tolerance. We also found that IFN-γ production by group 1 ILCs has to be intrinsically regulated by GR to allow this IL-10–dependent host resistance to inflammatory disease.

Altogether, these data support a model in which HPA axis activation leads to GC production during an initial phase of inflammation induced by bacterial infection, which in turn controls IFN-γ production by group 1 ILCs in spleen and liver via GR. The respective roles of NK cells and ILC1s in this process are difficult to differentiate in the absence of an available model for selectively depleting one of these subpopulations. However, based on the frequency of each cell type and the fact that ILC1s are susceptible to this regulation by GR only during the priming phase, we favor a model in which NK cells have a more prominent role. Nevertheless, this regulatory mechanism is required for the establishment of a state of immunosuppression characterized by high serum concentrations of the anti-inflammatory cytokine IL-10 and resistance to septic shock.

### Concluding remarks

The ability of host organisms to tolerate, at least to some extent, the presence of a pathogen can be seen as part of a global defense strategy enabling the host organism to protect itself from infectious diseases not only by fighting against pathogens but also by reducing the negative impact of infection on host fitness (Medzhitov et al., 2012). The HPA-dependent pathway controlling IFN-γ release by spleen and liver group 1 ILCs described here reveals an important mechanism of the fine balance facilitating the activation of the immune response by an infectious agent while preserving host integrity, which could be integrated into the broader concept of “disease tolerance.” This pathway plays a role upstream from myeloid cell reprogramming, the principal process investigated to date, and is essential for the development of an immune suppressive state, in which IL-10 is a major player.

In some clinical circumstances, the refractory state that allows resistance to endotoxin may also be associated with an increase in susceptibility to secondary nosocomial infections (Cavaillon et al., 2003). A loss of LPS reactivity similar to that reported in patients with sepsis has been found in patients with noninfectious systemic inflammation response syndrome (SIRS) caused by trauma, surgery, or cardiac arrest, who have a higher risk of succumbing to infections than other patients. Interestingly, in blood samples from patients with bacterial sepsis or SIRS, IFN-γ production in response to TLR agonists is abolished (Souza-Fonseca-Guimaraes et al., 2012a,b). In addition, NK cell immunosuppression has been observed in blood samples from trauma patients with brain injury, where poor NK cell recruitment into a BCG-induced granuloma model was observed (Deknuydt et al., 2013). Because the HPA axis can also be activated in such stressful situations, a knowledge of the molecular and cellular mechanisms involved in the induction of this state of tolerance may be important for the development of strategies for restoring a functional immune response in these patients. Our present findings thus shed new light on the critical role of group 1 ILCs in inflammation and the regulatory mechanisms promoting disease tolerance, paving new avenues of translational research for the treatment of inflammatory disorders.

## Materials and methods

### Mice

C57BL/6J mice were purchased from Janvier Labs; *Ncr1^iCre^* mice were generated as described previously (Narni-Mancinelli et al., 2011), and *Nr3c1^LoxP/LoxP^* mice were provided by F. Tronche (Sorbonne Universités, Université Pierre et Marie Curie, UMR_CR18, Neuroscience, Paris-Seine, Paris, France; Tronche et al., 1999). All mice were bred and maintained under specific-pathogen–free conditions at the Centre d’Immunophenomique de Marseille and in the Centre d’Immunologie de Marseille Luminy. Mice were housed under a standard 12 h/12 h light–dark cycle with food and water ad libitum. Age-matched (7–12 wk old) and sex-matched littermate mice were used. All experiments were conducted in accordance with institutional committees (Comité d’éthique de Marseille 14) and French and European guidelines for animal care.

### Flow cytometry

Single-cell suspensions from the spleen or liver (after lymphocyte isolation on a 37.5%-67.5% Percoll gradient) or small intestine (after digestion with collagenase VIII from Sigma and lymphocyte isolation on a 40–100% Percoll gradient) were incubated with Fc blocking antibody (2.4G2) and the fixable blue dead cell stain kit (Invitrogen). Surface molecules were stained using antibodies against CD45.2 (104), CD3 (145-2C11), CD19 (1D3), NK1.1 (PK136), CD49a (Ha31/8), CD11b (M1/70), CD27 (LG.3A10), CD43 (S7), KLRG1 (2F1), Ly49G2 (4D11), CD69 (H1.2F3), CD11b (M1/70), MHCII (M5/114.15.2), Ly6C (AL-21), CD3 (145-2C11), CD19 (1D3) from BD Biosciences; NKp46 (29A1.4), CD49b (DX5), NKG2A (16a11), Ly49D (4E5), Ly49H (3D10), and F4/80 (BM8) from eBioscience and CD11c (N418) and Ly6G (1A8) from BioLegend. For intracellular staining, cells were fixed and permeabilized with an intracellular staining kit (eBioscience) and the following antibodies were used: anti–GR XP rabbit mAb (D8H2) and rabbit mAb IgG XP (DA1E) from Cell Signaling Technology; anti–IFN-γ (XMG1.2) from BioLegend; anti–Rorγt (Q31-378), anti-TNF (MP6-XT22), anti–granzyme B (GB11), anti-Ki67 (B56), and anti–IL-10 (JE5-16E3) from BD Biosciences; and anti-Eomes (Dan11mag) from eBioscience.

### In vitro splenocyte stimulation

Splenocytes from control and GR*^Ncr1-iCre^* mice were stimulated in vitro with 25 ng/ml IL-12 and 20 ng/ml IL-18 in complete culture medium (RPMI 10% FCS, 100 µg/ml penicillin/streptomycin, 2 mM l-glutamine, 1 mM sodium pyruvate, and 0.01 M Hepes) with the addition of 500 nM corticosterone (Sigma; dissolved in ethanol) or the same volume of vehicle alone. Cells were stimulated at 37°C in the presence of Golgi Stop and Golgi Plug from BD Biosciences. After 4 h of stimulation, the cells were washed and stained for FACS analysis.

### Ex vivo cell stimulation

For IL-10 detection, splenocytes were stimulated with 500 ng/ml PMA and 500 ng/ml ionomycin (both from Sigma) for 2 h at 37°C in the presence of Golgi Plug. For IFN-γ detection in small intestine lymphocytes, cells were stimulated with 200 ng/ml PMA, 1 µg/ml ionomycin, 25 ng/ml IL-12, and 20 ng/ml IL-18 for 2 h at 37°C in the presence of Golgi Stop and Golgi Plug.

### Serum analysis

Blood was collected from the retro-orbital sinus of mice receiving LPS injections under low-stress conditions (i.e., within 2 min of handling). Sera were tested with the Corticosterone ELISA kit (Enzo) according to the manufacturer’s instructions for determining corticosterone concentration. The concentrations of IL-6, TNF-α, IFN-γ, and IL-10 were assessed with cytometric bead arrays according to the manufacturer’s protocol (CBA; BD Biosciences).

### In vivo LPS challenge

LPS (from *Escherichia coli* strain 055:B5; Sigma) diluted in PBS was injected intraperitoneally (20 µg per gram mouse body weight), and in endotoxin tolerance experiments, 5 µg LPS was injected, in a total volume of 30 µl, into the footpads of mice under anesthesia with 3.5% isoflurane. All LPS injections were performed between 9 a.m. and 10 a.m., and mice were checked every 12 h for signs of distress in survival experiments. Cytokine neutralization was achieved by the intraperitoneal injection of 500 µg anti–IFN-γ (XMG1.2), anti–IL-10 (JES5-2A5), or rat IgG1 (HRPN; all from BioXCell) at the time of LPS priming.

### Statistical analysis

Statistical analysis was performed with GraphPad Prism software. Data were considered statistically significant if the p-value obtained was lower than 0.05. Data were compared with unpaired Student’s *t* tests if the values followed a Gaussian distribution with similar variances or the Mann–Whitney *U* test otherwise. For multigroup comparisons, we applied one-way ANOVA or multiple *t* tests. Differences in survival were evaluated with the Mantel–Cox test.

### Online supplemental material

Fig. S1 shows that GR*^Ncr1-iCre^* mice have normal NKp46^+^ cell phenotype, numbers, and maturation. Fig. S2 shows that GR expression does not affect parameters linked to NKp46^+^ cell activation and functional competence other than IFN-γ production by group 1 ILCs in the spleen and liver in endotoxin tolerance. Fig. S3 shows that the low systemic IL-10 concentration in GR*^Ncr1-iCre^* mice is not directly caused by NKp46^+^ cell-intrinsic regulation by GR and is specific to endotoxin tolerance.

## Supplementary Material

Supplemental Materials (PDF)
