# Supplementary material for: Host resistance to endotoxic shock requires the neuroendocrine regulation of group 1 innate lymphoid cells
Source: J Exp Med. 2017 Dec 4;214(12):3531–41. doi: 10.1084/jem.20171048 (PMC5716043; doi:10.1084/jem.20171048)
Supplement: Supplemental Materials (PDF) [file JEM_20171048_sm.pdf]

The Journal of Experimental Medicine

SUPPLEMENTAL MATERIAL

Quatrini et al., https://doi.org/10.1084/jem.20171048

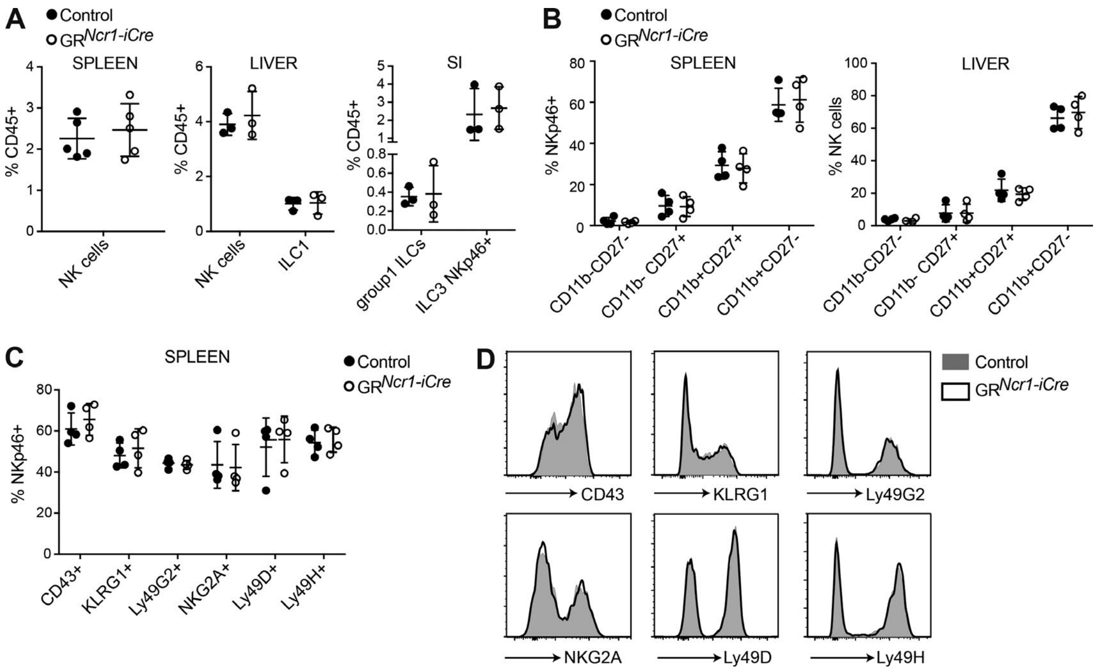

Figure S1. **Normal phenotype, number, and maturation of NKp46+ ILCs in GR$^{Ncr1\text{-}iCre}$ mice.** (A) Percentages of CD45$^+$ NK cells (NK1.1$^+$NKp46$^+$DX5$^+$CD49a$^-$), ILC1s (NK1.1$^+$NKp46$^+$DX5$^-$CD49a$^+$ in the liver), and, in the SI, group 1 ILCs (NK1.1$^+$NKp46$^+$) and ILC3s (CD3$^-$CD19$^-$Rorγt$^+$NKp46$^+$). Percentage of spleen (B and C) and liver (B) NK cells expressing the indicated markers at steady state. The data are presented as mean ± SD. Each symbol represents a single mouse. (D) FACS histograms showing spleen NK cell expression profile of the same markers as in C. Each histogram is representative of four mice per group.

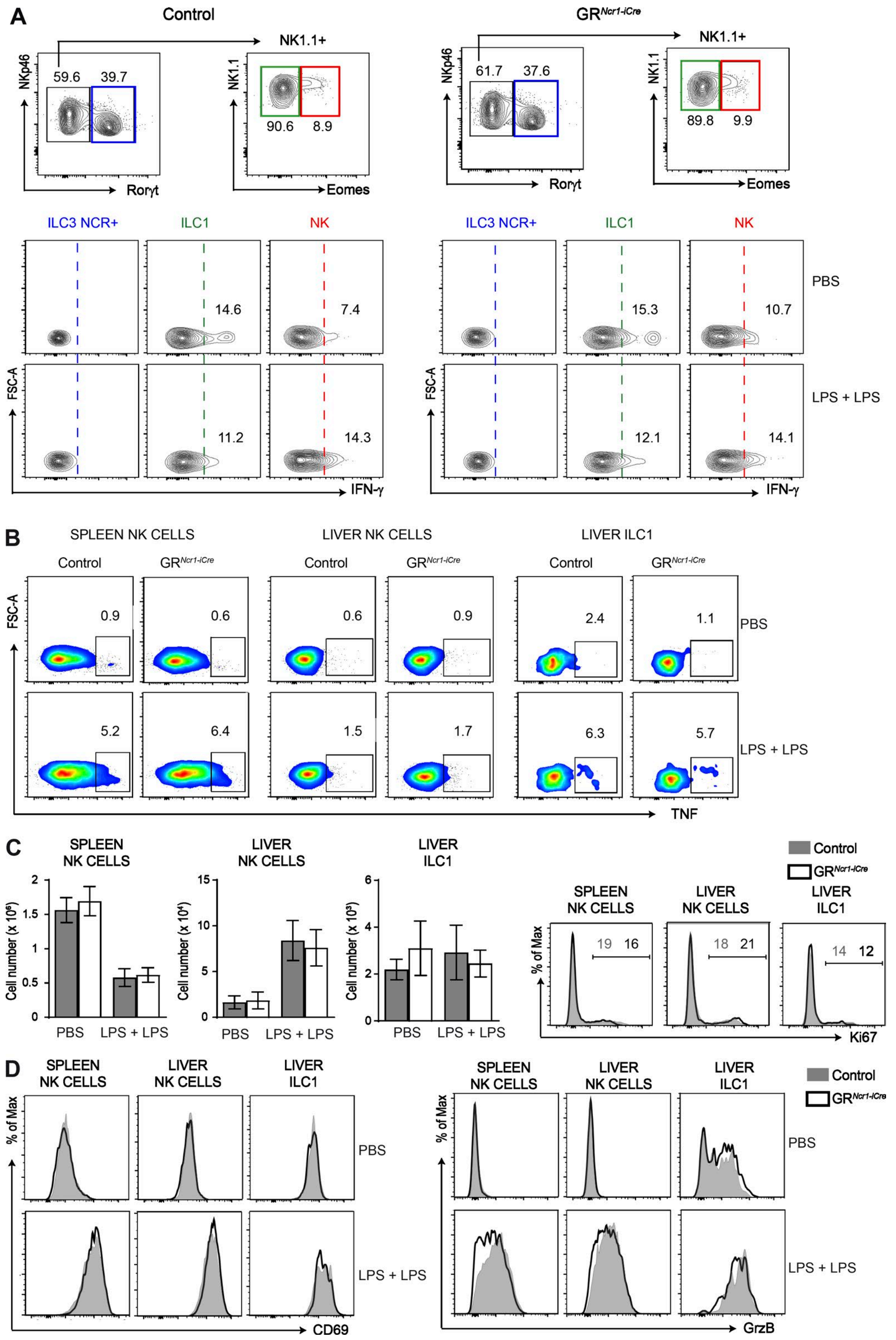

Figure S2. **Role of GR expression on the activation of NK cells and ILC1s in the context of endotoxin tolerance.** (A) Lymphocytes were isolated from the small intestine 6 h after LPS challenge and restimulated ex vivo for 2 h with PMA, ionomycin, IL-12, and IL18. IFN-γ staining in NKp46^{+} cells is shown after gating on CD45^{+}CD3^{-}CD19^{-} cells. TNF staining (B), cell numbers, Ki67 (C), and CD69 and granzyme B (D) staining in group 1 ILCs from the spleen and liver 6 h after LPS challenge. FACS plots show data from one representative experiment with three mice per group; in C, the data are shown as the mean ± SEM of two independent experiments with five or six mice per group.

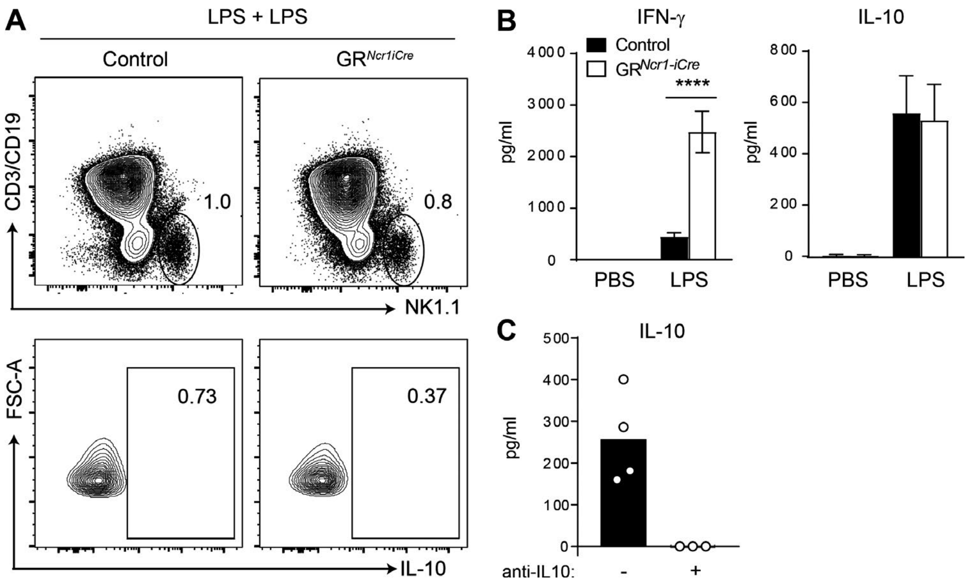

Figure S3. **The lower systemic IL-10 concentration in GR$^{Ncr1-iCre}$ mice is not caused by NK cell–intrinsic regulation by GR and is specific to endotoxin tolerance.** (A) FACS plots showing IL-10 intracellular staining in spleen NK cells 6 h after challenge with LPS. Data are representative of two independent experiments with seven mice per group. (B) Cytokines in the serum of mice 6 h after PBS or 20 µg/g LPS injection. Data are presented as mean ± SEM (n = 4–10 mice from two independent experiments; ****, $P < 0.0001$, Student's t test). (C) IL-10 levels in the serum of control mice receiving LPS injections and treated with anti–IL-10 neutralizing antibody during the priming phase, according to the protocol shown in Fig. 5 A.
